# The influence mechanism of community-built environment on the health of older adults: from the perspective of low-income groups

**DOI:** 10.1186/s12877-022-03278-y

**Published:** 2022-07-16

**Authors:** Zhenhua Zheng, Wanting Liu, Yingchen Lu, Ning Sun, Yusu Chu, Hong Chen

**Affiliations:** 1 College of Communication and Art Design, University of Shanghai for Science and Technology, Shanghai, China; 2 College of Architecture & Environment, Sichuan University, Chengdu, China

**Keywords:** The health of older adults, Community-built environment, Social participation, Outdoor exercise, Low-income groups

## Abstract

**Background:**

With the rapid development of population ageing, the international community has been paying more attention to the health problems of older adults and the age-friendly community. But there has not been enough discussion about the internal mechanism of the community-built environment that influences the health of older adults. The aim of our study was to explore the complex relationships among community-built environment, social participation, outdoor exercise, and health of older adults, as well as the differences among older adults in different income groups, particular attention was paid to the situation of low-income group.

**Methods:**

This study used descriptive statistical analysis and structural equation Modeling (SEM) to make a group comparison among older adults in different income groups. The data of this study came from a sample survey in Shanghai, China.

**Results:**

The study found that health difference exists among older adults in China: the lower the income, the worse the community-built environment, the worse the health. The community-built environment had an important impact on the health of older adults, especially the low-income older adults. And the community-built environment influenced the health of older adults through the intermediary role of outdoor exercise and social participation. Furthermore, the lower the income level of older adults, the stronger the direct effect of the community-built environment on their health; the higher the income level of older adults, the stronger the mediating effect of outdoor exercise and social participation on the impact of the community-built environment on their health.

**Conclusion:**

Governments should pay more attention to the health and living conditions of low-income older adults and take proactive steps to help them. Community design and construction should pay more attention to the demands of low-income older adult groups, which will help to improve the health inequality of older adults, consequently enhancing older adults’ overall health.

## Background

Population ageing is the current global population problem. The health problems of older adults not only affect the solution such as pensions and economic expenditures but also determine the healthy status and quality of life in each country and even the global population. Since the publication of *The Black Report* [1, 2], health inequalities have been an important topic in the international academic community [3–5]. Health disparities among different social groups exist objectively. The health status of higher socioeconomic groups is often better than that of lower socioeconomic groups, which is the phenomenon of health inequality [6–8]. The health equality of older adults has also been paid more attention with the accelerated speed of population ageing. Therefore, the health issues of vulnerable older adult groups deserve more attention, especially low-income group.

From “successful aging” to “healthy aging” to “active aging”, the concept of the international community to deal with population ageing has changed. The role of older adults should be changed from “passive recipients of support” to “active participants in social activities” [9]. Then the concept of “age-friendly communities” emerges [10]. The ultimate objective of “age-friendly communities” is to promote more social activities and improve the health status of older adults [11, 12]. As the community optimization construction has the characteristics of intervention ability and implement ability, it is of great practical value to the construction of the aged-friendly community and the realization of active aging [13]. The community is the basic activity place and living space for residents. As a result, constructing a community-built environment that can effectively improve the health of various social groups has become the key to promoting social equity and improving the health of whole people. With the development and practice of age-friendly communities, the international community, scholars, and governments pay more and more attention to how the community-built environment affects the health of older adults [14–16].

Scholars have extensively studied the relationship between the community-built environment and the health of older adults [17–21]. With the development of related research, more and more scholars believe that it is difficult to explain the mechanism of the interaction between the community environment and the residents’ health clearly by only studying the influence of environment on health [22]. Even more, this approach may be biased by the absence of important variables. Therefore, based on the social ecology, the application of theories and methods in related research has been increasingly recognized and emphasized [11, 19]. It emphasizes the joint role of community environment and behavior in the ecological system that affects the health of residents [23]. Social ecology emphasizes the interaction of the environment, human behavior, and health. The impact of the community-built environment on older adults’ health is not isolated. The community environment affects older adults’ the lifestyle and behavior, and ultimately affects their health. Social ecology provides a good theoretical basis for a better understanding of the underlying mechanisms by which the community-built environment affects the older residents’ health through affecting their behavior. Previous studies paid more attention to outdoor exercise as an intermediary variable of the community-built environment affecting the health of older adults [24–26]. At present, there are few studies on social participation as an intermediary variable. However, in 2002, WHO has put forward the three-dimensional pillars of active ageing: health, participation and security in “Active ageing: a policy framework” [9]. As social participation is of great significance to active aging [9, 27, 28], it also gets increased attention [20, 21, 29, 30]. Nevertheless, the complicated interaction between the community-built environment, social participation, outdoor exercise, and older adults’ health has not been adequately discussed.

Furthermore, in the context of the rapid development of population ageing, if we want to build an “age-friendly community”, we must not ignore the difference between older adult groups [31, 32], and not design and construct the community-built environment by the homogeneous way [31, 32]. To improve the overall health level of older adults, we must recognize the diverse demands of different older adult groups. Due to the significant differences in living conditions, behavioral habits and psychological needs of older adults at different income levels, the factors affecting their health are also different. Therefore, the comparative study about the effect of the community-built environment on the health of different income older adult groups will helps to promote the overall older adults’ health.

In recent years, China has been facing the biggest and fastest-growing population ageing process, which includes a large number of low-income groups [33]. Our research focuses on the community-built environment and health of the older adults in China, especially the situation of low-income groups. The aim of our study was to explore the complex relationships among community-built environment, social participation, outdoor exercise, and health of older adults, as well as the differences among older adults in different income groups, particular attention was paid to the situation of low-income group. Our research primarily raises the following questions based on the above literature review and analysis:

1. Are there significant differences in health status, behavior and community-built environment among older adults with different income levels? Is there health inequality?
2. Does the community-built environment have significant influence on older adults’ behavior and health? Whether the behavior pattern is the intermediary variable of the community-built environment affecting the health of older adults?
3. Are there significant differences in the way of community-built environment on the health of older adults with different income? What are the characteristics of low-income groups?

## Method

### Study population

**Fig. 1:**
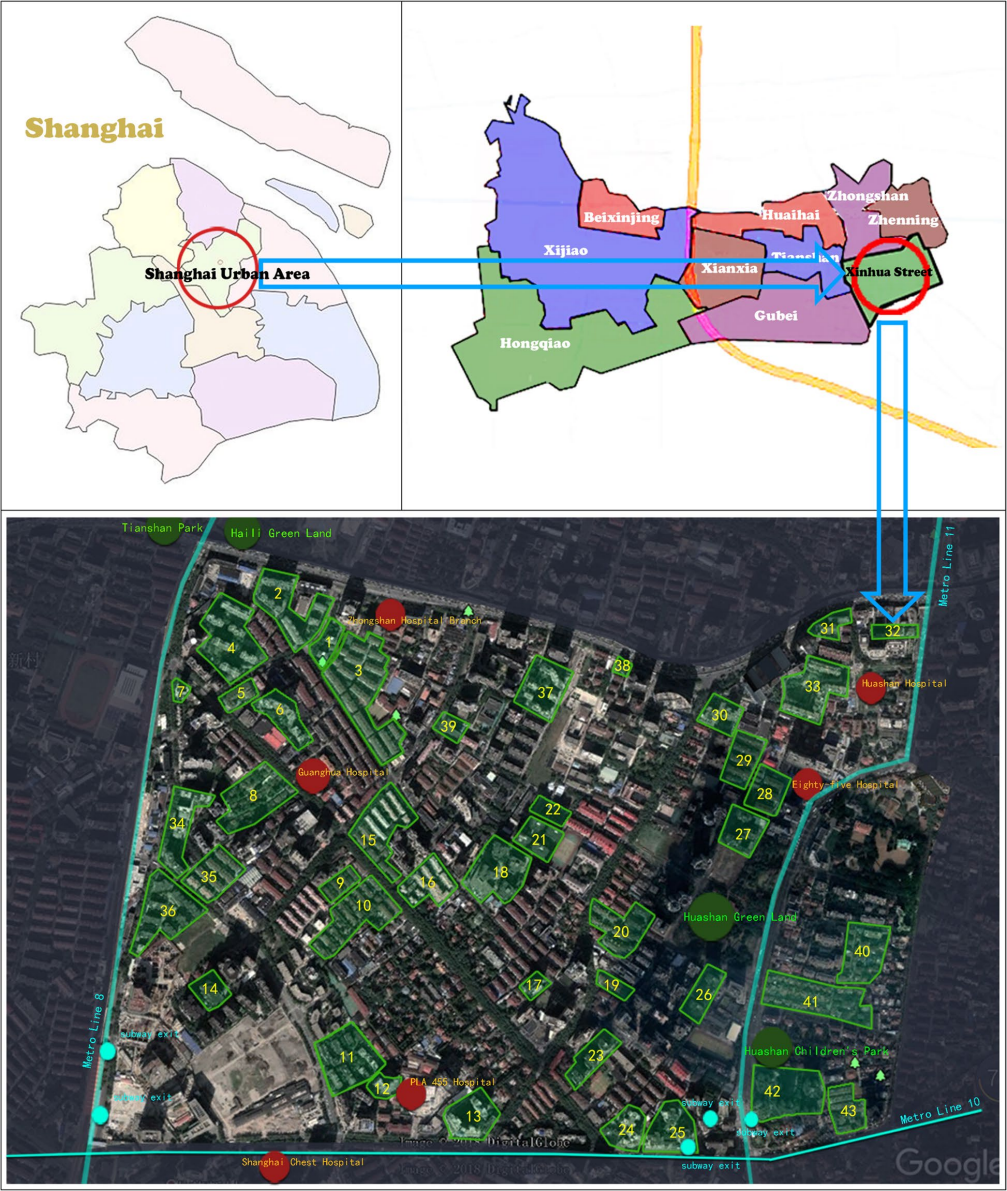
Map of Xinhua Street the community sample

**Fig. 2:**
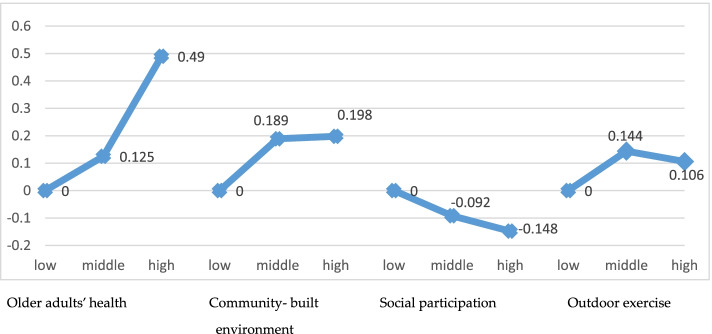
Comparison of the community-built environment, social participation, outdoor exercise, and health differences between older adults with different incomes

The data for this study came from a large sample survey conducted by Public Health College of Fudan University. The purpose of the survey was to investigate the relationship between the community-built environment social participation, outdoor exercise and health of older adults. This research place was selected in Xinhua Street, Changning District, Shanghai (Fig. 1). The Xinhua Street is located in the southeast of Changning District, which is in the center of Shanghai. It covers an area of 2.2 km^2^ and has 22,000 households and a population of about 85,000, of which 16% are over 65 years old. Xinhua Street has a long history and a variety of community types. Xinhua Street has communities built in different periods from 1930s to 2010. From villa communities to old apartments with poor environmental facilities, Xinhua Street had communities with different environmental quality. In addition, the housing unit price of the communities in Xinhua Street are also very different, ranging from 60,000 to 200,000 yuan per square meter. All of these can reflect the residents of different communities in Xinhua Street within the income gap. The survey used a two-stage sampling method from June 2014 to October 2014. First, we selected 43 out of 198 communities. To make the sample as representative as possible, community samples should be selected as far as possible to cover different quality communities in Xinhua Street. Geographical location, convenient transportation, and construction age often have great significance in explaining the quality of the community. Therefore, this survey’s primary sampling principle was based on these aspects. See Fig. 2 for details. Then, in the selected community, a sample survey of older adults aged 60 and over without cognitive impairment was conducted. The sample principle was if the number of older adults in selected community was less than 120, all of them were surveyed; if the number of older adults in selected community was more than 120, 120 older adults were chosen at random. The list of older adults without cognitive impairment was provided by the neighborhood committee, and 2783 valid samples were obtained. There were 1292 low-income older adults’ samples, 964 middle-income older adults’ samples and 527 higher-income older adults’ samples.

### Measurement

#### Dependent variable: the health of older adults

Self-evaluation of health has been widely used in self-perceived of overall health [34, 35], which is highly predictive of functional disability, morbidity and mortality [36] and is even more important than actual medical measurements results [37]. So, self-evaluation of health was thought to be an excellent predictor of objective health status [38]. This paper used self-rated health and health satisfaction to assess the health of older adults. On a scale of 1 to 10, the higher the score, the better the health.

#### Independent variable: community-built environment

The community-built environment included the leisure environment and the landscape environment. They were measured using two measurement models of community-aware environment developed by Mujahid et al. [39]. The leisure environment included seven dimensions: walking convenient, walking fitness, sufficient trees, exercise opportunities, sports facilities, walking attraction and exercise attraction. And the landscape environment included three dimensions: the interest of architecture, environment cleanliness and the attraction degree. The responses to each item ranged from 1 to 5 (1 = completely, 2 = disagree, 3 = neutral, 4 = completely, 5 = agree) and the higher score indicated higher degree of acceptance of the leisure environment and the landscape environment.

#### Intermediary variables: social participation, outdoor exercise

The social participation of older adults in this paper mainly referred to the activities of older adults in the community, included five activities: volunteer work, self-management and mutual assistance activities, lectures and reports, participation in cultural activities, and participation in interest groups. Older adults’ level of social participation was assessed by asking them about the frequency of participated in various activities over the past 12 months. The item was scored on a scale 1 to 5(1 = never, 2 = several times a year, 3 = several times a month, 4 = once a week, 5 = 2–3 times a week), with the higher score indicating more social participation.

Outdoor exercise included two observation variables: walking frequency and walking duration. The walking frequency was measured by the number of walking per week. The walking duration was the about time of each walk.

#### Control variable

Age, gender, education and community residence time were included as the control variable in this paper’s conceptual model. Gender as two categorical variables, male as 0 and female as 1. Education levels were assigned as follows: 1 = junior high school and below, 2 = senior high school, technical secondary school and technical school, 3 = junior college, 4 = bachelor, 5 = master and above.

### Statistical analysis

This study analyzed how the community-built environment affect the health of older adults through outdoor exercise and social participation as the intermediary. Structural Equation Modeling (SEM) was more suitable for analyzing this complex relationship. The SEM had the benefits of visualization, intuition, and science in dealing with the comparative analysis of multi-group models. So, this paper used SEM analysis and Maximum likelihood estimation method. The structural equation model was fitted using MPLUS software. The processing of the mean of latent variables was one advantage of the SEM. Unlike other statistical methods that add the mean to latent variables, the structural equation model was systematically analyzed by the different weights of each measured variable and eventually appears as difference among the means of variables in different groups. MPLUS set the low-income group to 0 and used software analysis to determine the specific difference between middle-income group, high-income group, and low-income group. The use of a SEM latent mean comparison allowed for a more precise measurement of the differences in variable means between various income groups.

Multi-factor confirmatory analysis was performed on all the measurement models in the conceptual model, and the compositional reliability of all the measurement models was greater than 0.6; the average variance extraction was greater than 0.5; the factor load of the observed variables was greater than 0.6; the reliability coefficient was greater than 0.36 [40]. All the measurement models had good reliability and validity. The leisure environment and landscape community walking support environment had a correlation coefficient of 0.632. Therefore, the leisure environment and the landscape environment constituted the second-order model of the community-built environment.

The results of model fitting demonstrated that the CFI did not achieve the ideal standard, indicating that the model had to be improved. After establishing the converged relationship between “sport facilities” and “exercise facilities”, “interesting design” and “attractive”, and “attractive” and “clean and tidy”, the final IFI, CFI and X2/DF all achieved the criteria, thereby the optimized model was fit. The final indexes (CFI > .90, TLI > .90, RMSER<.08) achieved the criteria, which show that the model was fit. The model fit indicators are shown in Table 1.

**Table 1:** Model fit indicators

	CFI	IFI	TLI	CFI	RMSEA	X^2^/DF
Whole model	0.954	0.957	0.947	0.965	0.045	3.891
Low-income model	0.918	0.919	0.905	0.978	0.060	1.889
Middle-income model	0.940	0.941	0.930	0.978	0.052	2.706
High-income model	0.947	0.949	0.939		0.050	2.320
Ideal standard	> 0.9	> 0.9	> 0.9	> 0.9	< 0.08	< 5

## Results

### Descriptive statistics

The descriptive statistics of variables in Table 2 reveal that older adults’ health satisfaction is greater than self-rated health. It indicates that the whole older adults have a better mentality. Self-rated health and health satisfaction improve with income growth. In the community-built environment, the average of the leisure environment is generally greater than that of the landscape environment. The mean value of all measurement variables in the community-built environment reflects that the low-income older adults have lower than middle-income and high-income older adults. The level of older adults’ social participation is very low, and the average value of all participation activities is below 2, implying that the frequency of older adults’ social participation is generally several times a year. Older adults’ walking frequency is 4.2 times a week with a walking time of 28.57 minutes. Their walking frequency and time increase as income increases. In the control variables, the average age of older adults are 72.7 years, with a balanced gender structure, and the overall education level is above senior high school, having a more than 22 years’ living time on average in their community. With rising income, the average age of the older adults gradually decreases, the level of education gradually increases, the number of males gradually increases, and the living time in their community shortens progressively.

**Table 2:** Variable descriptive statistics

		Observed variable		sample							
				The total		Low income		Middle income		High income	
				Mean	Std. deviation	Mean	std. deviation	Mean	std. deviation	Mean	std. deviation
Health of older adults		SRH	Self-rated health	2.35	0.782	2.22	0.791	2.26	0.761	2.52	0.778
		H_S	Health satisfaction	3.61	0.960	3.38	0.925	3.56	0.958	3.80	0.948
Community-built environment	Walking environment	WE1	Walking attraction	3.32	1.031	3.17	0.996	3.30	0.953	3.39	1.130
		WE2	Exercise attraction	3.18	1.071	2.99	1.051	3.18	0.981	3.26	1.176
		WE3	Sports facilities	2.95	1.049	2.76	1.136	2.93	0.969	3.04	1.088
		WE4	Exercise opportunities	3.00	1.056	2.73	1.190	3.01	0.956	3.09	1.085
		WE5	Enough trees	3.14	0.974	3.09	0.920	3.16	0.916	3.13	1.067
		WE6	Walking convenience	3.65	0.923	3.60	0.865	3.68	0.889	3.65	0.990
		WE7	Suitable for walking	3.31	0.972	3.22	0.946	3.31	0.917	3.34	1.047
	Sensory environment	SE1	Interesting design	2.72	0.820	2.50	0.880	2.74	0.819	2.78	0.773
		SE2	attractive	2.92	0.834	2.66	0.947	2.95	0.806	2.99	0.788
		SE3	cleanliness	2.87	0.890	2.69	0.874	2.85	0.830	2.95	0.953
Social participation		SP1	Interest groups	1.87	1.327	1.93	1.416	1.78	1.283	1.94	1.330
		SP2	Community activities	1.76	1.262	1.75	1.305	1.68	1.251	1.76	1.256
		SP3	Lecture report	1.65	0.936	1.65	0.973	1.67	0.964	1.62	0.881
		SP4	Mutual assistance groups	1.50	0.972	1.55	0.991	1.53	0.999	1.42	0.972
		SP5	volunteer	1.64	1.069	1.63	1.117	1.65	1.116	1.50	0.972
Outdoor exercise		OE1	Walking frequency	4.20	2.904	4.08	2.874	4.19	2.938	4.26	2.879
		OE2	Walking time	28.57	33.588	24.85	22.470	29.67	31.237	29.02	40.217
Control variable		age		72.74	7.809	80.62	8.076	71.43	8.119	70.74	7.268
		gender		0.58	0.493	0.687	0.464	0.61	0.488	0.49	0.500
		education		2.25	1.069	1.76	0.872	1.90	0.901	2.90	1.023
		Living time		1.87	15.540	26.44	17.787	25.13	16.459	16.35	10.764

The difference in variable mean of different income groups is shown in Fig. 2. With the increase in income level, the health status of older adults and the community-built environment shows a trend of gradual improvement, especially the health status of older adults show a larger increase. The health status of older adults with high and middle income is higher than that of older adults with low income, and the difference values are 0.125 and 0.490, respectively. And the difference values in the community-built environment are 0.189 and 0.918, respectively. According to the study’s findings, there are significant differences in the health of older adults with different incomes. As a result, it is essential to discuss the health path of different income older adult groups. Older adults’ frequency of social participation activities gradually reduces as their income level increases, and the difference values are − 0.92 and − 0.148, respectively. The outdoor exercise shows an inverted V-shaped relationship, with middle-income older adults having the highest intensity and low-income older adults having the lowest intensity. The difference values in outdoor exercise are 0.144 and 0.106, respectively.

### Analysis based on the models of full sample

**Fig. 3:**
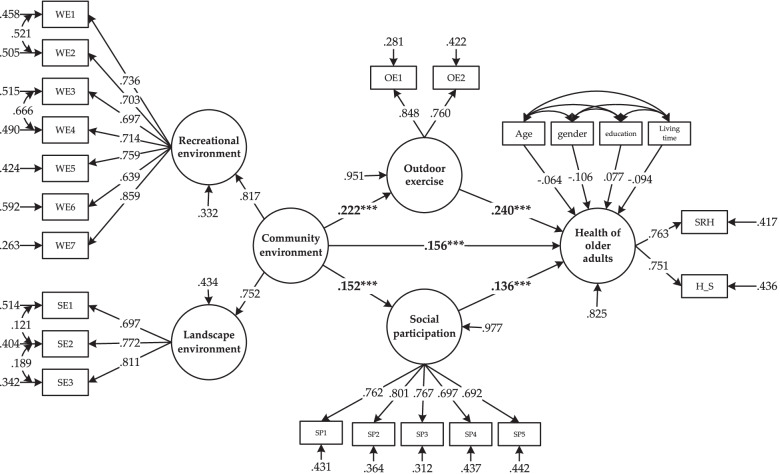
The standardization coefficient of the path of the overall model for older adults

The model-fitting results based on the entire sample are shown in Table 3 and Fig. 3. After controlling for age, gender, education, and community living time, the total effect value for the community-built environment, social participation, and outdoor exercise on the health of older adults are 0.230, 0.136, and 0.240, respectively.

**Table 3:** Total, direct and indirect effects of the overall model path

Independent variable	Intermediate variable		Dependent variable		
	social participation	Outdoor exercise	older adults’ health		
			Total effect	Direct effect	Indirect effect
**Community-built environment**	0.152***	0.222***	0.230***	0.156***	0.074***
**Social participation**	–	–	0.136***	0.136***	–
**Outdoor exercise**	–	–	0.240***	0.240***	–

Note: *** represents significant at the 1% level

** represents significant at the 5% confidence level

* represents significant at the 10% confidence level

The direct and indirect effects of the community-built environment on older adults’ health are significant, indicating some intermediary variables in the path. The intermediary effect values of social participation and outdoor exercise are 0.021 and 0.053, respectively. It means that the positive impact of the community-built environment on older adults’ health needs to be realized by promoting outdoor exercise and social participation.

### Comparison of model differences among different income groups

**Fig. 4:**
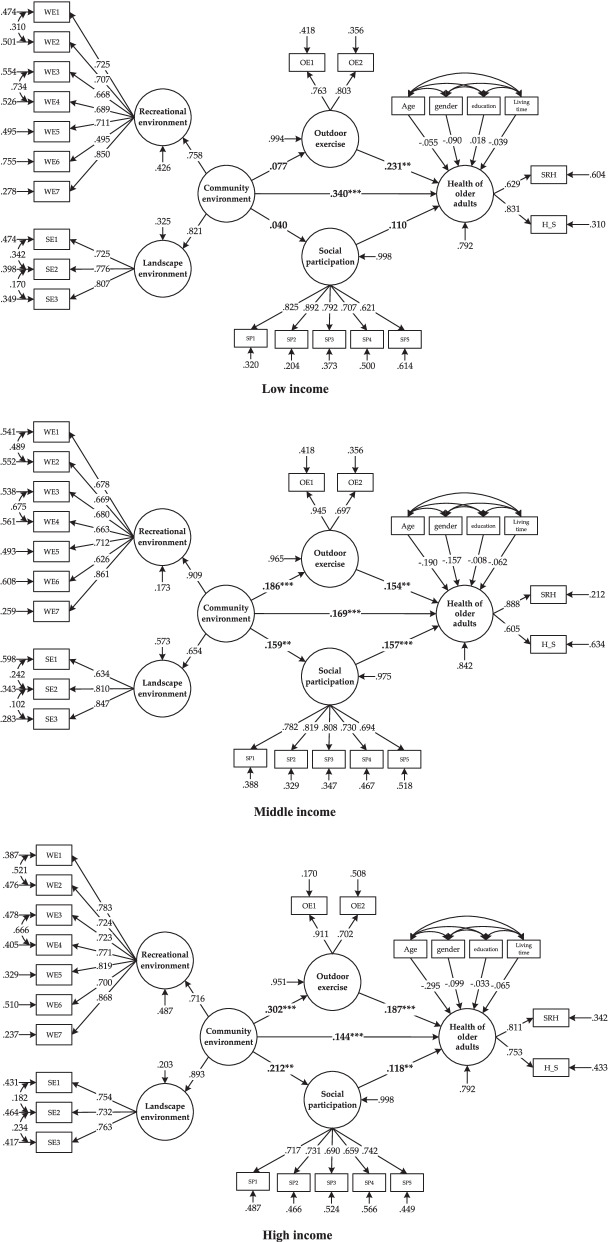
Comparison of the path of the model for older adults with different incomes

We compared the model path of different income older adult groups, and the output results showed that the path coefficient was set to the same *P*-value< 0.05, indicating significant differences in group model paths of different income levels. The model-fitting results based on different income groups are shown in Table 4, Figs. 3 and 4.

**Table 4:** Comparison of different income older adults model paths

Independent variable		Intermediate variable		Dependent variable		
		social participation	Outdoor exercise	older adults’ health		
				Total effect	Direct effect	Indirect effect
**low income**	**Community-built environment**	0.040	0.077	0.362***	0.340***	0.022
	**Social participation**	–	–	0.110	0.110	–
	**Outdoor exercise**	–	–	0.231**	0.231**	–
**middle income**	**Community-built environment**	0.159**	0.186***	0.223***	0.169***	0.054**
	**Social participation**	–	–	0.157***	0.157***	–
	**Outdoor exercise**	–	–	0.154**	0.154**	–
**high income**	**Community-built environment**	0.212**	0.302***	0.225***	0.144***	0.081**
	**Social participation**	–	–	0.118**	0.118**	–
	**Outdoor exercise**	–	–	0.187***	0.187***	–

Note: *** represents significant at the 1% level

** represents significant at the 5% confidence level

* represents significant at the 10% confidence level

The total effect value of the community-built environment and outdoor exercise on the health of the low-income older adults are 0.340 and 0.231, respectively. On the other hand, social participation has no effect on them. The direct effect of the community-built environment on the low-income older adults’ health is significant, but the indirect effect is not significant. It indicates there is no intermediary effect in the path, which means the effect of the community-built environment on the low-income older adults’ health is direct and would not be interfered by outdoor exercise and social participation.

The community-built environment, outdoor exercise, and social participation all have significantly positive effects on the health of middle-income older adults, with effect values of 0.223, 0.157, and 0.154, respectively. The direct and indirect effects of the community-built environment on the health of middle-income older adults are significant, indicating a partial intermediary effect in the path. The intermediary effect value of social participation is 0.025, and the intermediary effect value of outdoor exercise is 0.029. It shows that outdoor exercise and social participation would help achieve the positive effect of the community-built environment on the health of middle-income older adults.

The community-built environment, outdoor exercise, and social participation all significantly positively affect the health of high-income older adults, with effect values of 0.225, 0.118 and 0.187, respectively. The direct and indirect effects of the community-built environment on the health of high-income older adults are significant, indicating a partial intermediary effect in the path. The intermediary effect value of social participation is 0.035, and the intermediary effect of outdoor exercise is 0.056. According to the total effect (0.225), direct effect (0.144), and indirect effect (0.081) of the community-built environment on the path of the high-income older adults, we can see that the indirect effect of outdoor exercise and social participation has reached 36%. These data mean that for high-income older adults, outdoor exercise and social participation play an essential intermediary role in how the community-built environment influences their health.

## Discussion

Our study explored the complex interaction among the community-built environment, social participation, outdoor exercise, and older adults’ health, as well as the difference between older adults in different income groups, especially the situation of low-income groups.

Our study confirmed the existence of the health inequality problem in older adults [6–8]. The higher the income, the better the health and the community-built environment. The lower the income, the worse the health and community-built environment. The research also discovered significant differences in the behavior of older adults with different income levels: the higher the older adults’ income, the lower the frequency of social participation. However, the outdoor exercise showed an inverted V-shaped relationship, with the highest outdoor sports intensity in middle-income older adults and the lowest outdoor exercise intensity in low-income older adults.

Our study confirmed the community-built environment significantly affect the health of older adults [11, 19–21, 31, 32]. Meanwhile, the community-built environment influenced the health of older adults through the mediation of outdoor exercise and social participation. That is, improving the quality of the community-environment would increase older adults’ frequency of outdoor exercise and social participation, then improve their health.

More importantly, our study found significant differences in the pathways by which community-built environments affect the health of older adults at different income levels. The lower the older adults’ income level, the greater the direct effect of the community-built environment on their health. The higher the older adults’ income level, the stronger the intermediary effect of outdoor exercise and social participation on the effect of the community-built environment on their health. The community-built environment had a strong and direct effect on the health of the low-income older adults, and outdoor exercise and social participation did not affect it. At the same time, the community-built environment through the intermediary effect of outdoor exercise and social participation affected the health of middle-income and high-income older adults.

The influence path of the community-built environment on the health of older adults with different income levels is different. Therefore, to truly improve the health of older adults and reduce health inequality, it is necessary to consider the needs of various income groups, with a particular focus on the characteristics of the low-income older adults, to achieve differentiated responses and accurate environmental governance. The community-built environment significantly influences the health of low-income older adults, with a total effect value of 0.362. Meanwhile, this effect is independent and will not be affected by outdoor exercise and social participation. In addition, the low-income older adult had the worst health status, and the community-built environment had the largest effect on the health of the low-income older adults. These implied that improving the low-income older adults’ community-built environment would enormously affect their health. Therefore, during the urban redevelopment process, relevant government departments and environmental designers should give special attention to improving the community-built environment for the low-income older adults.

However, the study still has some limitations. First, the survey scope and community sample quantity are limited. Since only the Xinhua community in the Changning district of Shanghai is selected for the in-depth survey, the research conclusion cannot represent all urban China community-built environments, and more empirical studies should be conducted in the future. Secondly, the representativeness of older adult samples requires further improvement. In selecting communities, although the current study takes geographical location diversity, transportation convenience and completion year as the sampling principles, it still fails to develop systematic random sampling. So, it needs to increase the uncertainty of older adults’ samples. Finally, the assessment of the community-built environment in this study is subjective. The following research should combine subjective and objective assessments of the community-built environment. It can better explore the association between the community-built environment and the health of older adults.

## Conclusion

The results showed that health inequality exists among older adults. The higher the income, the better the health. The lower the income, the worse the health status. As a result, extra attention must be paid to the health problems of low-income older adults.

Our study found that the community-built environment had a significant effect on the health of older adults, and outdoor exercise and social participation were intermediary variable for the community-built environment affecting the health of older adults. More importantly, we discovered differences in the effect of the community-built environment on the health of different income groups. The lower the income level of older adults, the stronger the direct effect of the community-built environment on their health. The higher the income level of older adults, the stronger the mediating effect of outdoor exercise and social participation on their health. We advise all governments to pay more attention to the health and the community-built environment of low-income older adults. We strongly suggest that, in the future planning, design, construction and renewal process of the community-built environment, we should pay more attention to the needs of low-income older adult groups and give them care. This suggestion would assist in reducing health inequality and thus improve the health of older adults.

## Data Availability

The datasets used and analysed during the current study available from the corresponding author on reasonable request.

## Abbreviations

SEM: Structural equation Modeling
CFI: Goodness-of-fit index
IFI: Incremental fit index
X2/DF: Chi-Square/Degree of freedom
RMSEA: Root Mean Square Error of Approximation

## References

[1] Black D, Morris J, Smith C, Townsend P (1980). Inequalities in health: a report of a research working group.

[2] Black D (1999). A black look at the independent inquiry into inequalities in health. J R Coll Physicians Lond.

[3] Marmot M (2005). Social determinants of health inequalities. Lancet.

[4] Scambler G (2012). Health inequalities. Sociol Health Illness.

[5] Zhou Z, Fang Y, Zhou Z (2016). Assessing income-related health inequality and horizontal inequity in China. Soc Indic Res.

[6] Braveman P (2006). HEALTH DISPARITIES AND HEALTH EQUITY: concepts and measurement. Annu Rev Public Health.

[7] Claussen B. Socioeconomic status and health. Int Encyclopedia Soc Behav Sci. 2015;18.

[8] Nuru-Jeter AM, Michaels EK, Thomas MD, Reeves AN, Thorpe RJ, LaVeist TA (2018). Relative roles of race versus socioeconomic position in studies of health inequalities: a matter of interpretation. Annu Rev Public Health.

[9] World Health Organization (2002). Active aging: a policy framework.

[10] World Health Organization (2007). Global age-friendly cities: a guide.

[11] Menec V, Nowicki S. Examining the relationship between communities’ “age-friendliness” and life satisfaction and self-perceived health in rural Manitoba, Canada. Rural Remote Health. 2014. 10.22605/rrh2594. 24437338

[12] Scharlach AE (2017). Aging in context: individual and environmental pathways to aging-friendly communities—the 2015 Matthew A. Pollack Award Lecture. Gerontologist.

[13] Plouffe L, Kalache A (2010). Towards global age-friendly cities: determining urban features that promote active aging. J Urban Health.

[14] de Vries S, Verheij RA, Groenewegen PP, Spreeuwenberg P (2003). Natural environments—healthy environments? An exploratory analysis of the relationship between Greenspace and health. Environ Planning A: Economy and Space.

[15] Xue XD, Cheng MM. A study on relationship of social capital, health and happiness among rural elderly in China——empirical analysis based on survey data in Hubei and Henan Province. Econ Manag J. 2012.

[16] Smith RJ, Lehning AJ, Dunkle RE (2013). Conceptualizing age-friendly community characteristics in a sample of urban elders: an exploratory factor analysis. J Gerontol Soc Work.

[17] Berke EM, Koepsell TD, Moudon AV, Hoskins RE, Larson EB (2007). Association of the Built Environment with Physical Activity and Obesity in older persons. Am J Public Health.

[18] Chen Y, While AE, Hicks A (2014). Self-rated health and associated factors among older people living alone in Shanghai. Geriatr Gerontol Int.

[19] Moore KD (2014). An ecological framework of place: situating environmental gerontology within a life course perspective. Int J Aging Hum Dev.

[20] Zheng Z, Yang L, (Lydia). Neighborhood environment, lifestyle, and health of older adults: comparison of age groups based on ecological model of aging. Sustainability. 2019;11(7). 10.3390/su11072077.

[21] Zheng C, Yang. (2019). Transfer of promotion effects on elderly health with age: from physical environment to interpersonal environment and social participation. Int J Environ Res Public Health.

[22] Wang R, Liu Y, Xue D (2019). Depressive symptoms among Chinese residents: how are the natural, built, and social environments correlated?. BMC Public Health.

[23] Oyeyemi AL, Kolo SM, Oyeyemi AY, Omotara BA (2018). Neighborhood environmental factors are related to health-enhancing physical activity and walking among community dwelling older adults in Nigeria. Physiother Theory Pract.

[24] Glicksman A, Ring L, Kleban M, Hoffman C (2013). Is “Walkability” A Useful Concept for Gerontology?. J Hous Elder.

[25] Wang Y, Chen YC, Shen HW, Morrow-Howell N (2017). Neighborhood and depressive symptoms: a comparison of rural and urban Chinese older adults. The Gerontologist.

[26] Joseph A, Zimring C (2007). Where active older adults walk. Environ Behav.

[27] United Nations Population Fund. Ageing in the 21st century: a Celebration and a challenge. Available online: https://www.unfpa.org/publications/ageing-twenty-first-century (accessed on 3 Apr 2019).

[28] Cramm JM, Nieboer AP. Social cohesion and belonging predict the well-being of community-dwelling older people. BMC Geriatr. 2015;15(1). 10.1186/s12877-015-0027-y. 10.1186/s12877-015-0027-y PMC4369354 25879773

[29] Rantakokko M, Iwarsson S, Kauppinen M, Leinonen R, Heikkinen E, Rantanen T (2010). Quality of life and barriers in the urban outdoor environment in old age. J Am Geriatr Soc.

[30] Lehning AJ, Smith RJ, Dunkle RE. Age-Friendly Environments and Self-Rated Health: Research on Aging. 2012;36(1):72–94. 10.1177/0164027512469214. 10.1177/0164027512469214 25651601

[31] Zheng Z, Chen H, Gao J (2021). Age differences in the influence of residential environment and behavior on the life quality of older adults: the transfer from physical-environment to social-behavior. Int J Environ Res Public Health.

[32] Zheng, Z.; Chen, H. The relationship among community environment, behavior, activity ability, and self-rated health of older adults: a hierarchical and multi-dimensional comparative study. Int J Environ Res Public Health 2021, 18, 7387. https://doi.org/10.3390/ijerph18147387 10.3390/ijerph18147387 PMC8304773 34299837

[33] Word Health Organization. Good health adds life to years: Global brief for World Health Day 2012. Retrieved from World Health Organization website: https://www.who.int/publications-detail-redirect/WHO-DCO-WHD-2012.2

[34] Jylhä M (2009). What is self-rated health and why does it predict mortality? Towards a unified conceptual model. Soc Sci Med.

[35] Pagotto V, Bachion MM, da Silveira EA (2013). Self-assessment of health by older Brazilians: systematic review of the literature. Rev. Panam. SaludPublica.

[36] Tsai AG, Boyle TF, Hill JO, Lindley C, Weiss K (2014). Changes in obesity awareness, obesity identification, and self-assessment of health: results from a statewide public education campaign. Am J Health Educ.

[37] Maddox GL, Douglass EB (1973). Self-assessment of health: a longitudinal study of elderly subjects. J Health Soc Behav.

[38] Wu S, Wang R, Zhao Y, et al. The relationship between self-rated health and objective health status: a population-based study. BMC Public Health. 2013;13(1). 10.1186/1471-2458-13-320. 10.1186/1471-2458-13-320 PMC3637052 23570559

[39] Mujahid MS, Diez Roux AV, Morenoff JD, Raghunathan T (2007). Assessing the measurement properties of neighborhood scales: from psychometrics to Ecometrics. Am J Epidemiol.

[40] Fornell C, Larcker DF (1981). Evaluating structural equation models with unobservable variables and measurement error. J Mark Res.

